# Prolonged outbreak of New Delhi metallo-beta-lactamase-producing carbapenem-resistant Enterobacterales (NDM-CRE), Tuscany, Italy, 2018 to 2019

**DOI:** 10.2807/1560-7917.ES.2020.25.6.2000085

**Published:** 2020-02-13

**Authors:** Lara Tavoschi, Silvia Forni, Andrea Porretta, Lorenzo Righi, Filippo Pieralli, Francesco Menichetti, Marco Falcone, Giulia Gemignani, Spartaco Sani, Paola Vivani, Tommaso Bellandi, Danilo Tacconi, Lucia Turini, Giulio Toccafondi, Gaetano Privitera, Pierluigi Lopalco, Angelo Baggiani, Fabrizio Gemmi, Grazia Luchini, Maurizio Petrillo, Lorenzo Roti, Patrizio Pezzotti, Annalisa Pantosti, Stefania Iannazzo, Maria Teresa Mechi, Gian Maria Rossolini, on behalf of the Tuscan Clinical Microbiology Laboratory Network

**Affiliations:** 1 Department of Translational Research and New technologies in Medicine and Surgery, University of Pisa, Pisa, Italy; 2 Regional Health Agency of Tuscany, Florence, Italy; 3 Quality of care and Clinical networks, Tuscany Region, Florence, Italy; 4 Florence Careggi University Hospital, Florence, Italy; 5 University Hospital of Pisa, Pisa, Italy; 6 Livorno Hospital, Toscana North-West Health Authority, Livorno, Italy; 7 Massa Carrara Hospital, Toscana North-West Health Authority, Massa Carrara, Italy; 8 Toscana North-West Health Authority, Lucca, Italy; 9 Arezzo Hospital, Toscana South-East Health Authority, Arezzo, Italy; 10 Toscana North-West Health Authority, Pisa, Italy; 11 Fondazione Toscana Gabriele Monasterio, Pisa, Italy; 12 Istituto Superiore di Sanità, Rome, Italy; 13 Ministry of Health, Rome, Italy; 14 Department of Experimental Medicine, University of Florence, Florence, Italy; 15 The members of the network are acknowledged at the end of the article

**Keywords:** Italy, outbreak, New Delhi metallo-beta-lactamase, Enterobacterales, Klebsiella pneumoniae

## Abstract

In Tuscany, Italy, New Delhi metallo-beta-lactamase-producing carbapenem-resistant Enterobacterales (NDM-CRE) have increased since November 2018. Between November 2018 and October 2019, 1,645 samples were NDM-CRE-positive: 1,270 (77.2%) cases of intestinal carriage, 129 (7.8%) bloodstream infections and 246 (14.9%) infections/colonisations at other sites. *Klebsiella pneumoniae* were prevalent (1,495; 90.9%), with ST147/NDM-1 the dominant clone. Delayed outbreak identification and response resulted in sustained NDM-CRE transmission in the North-West area of Tuscany, but successfully contained spread within the region.

An increase in isolates of NDM-producing carbapenem-resistant Enterobacterales (NDM-CRE) in samples obtained from patients admitted to hospitals in the North-West (NW) area of Tuscany has been registered since the last months of 2018 [1], leading, in March 2019, to the recognition by the Tuscany Regional Department of Health (RDH) of an outbreak situation and to the establishment of a multidisciplinary regional task force (RTF) to coordinate response activities.

Here we describe the spatial and temporal trend of NDM-CRE incidence in Tuscany since the emergence of the outbreak and the public health measures adopted.

## Prevention and response activities

**Figure 1:**
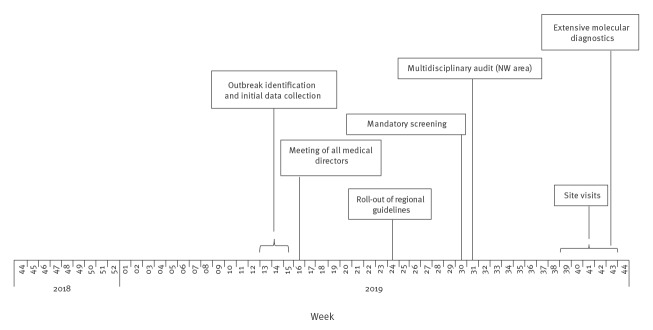
Actions to control the outbreak of NDM-CRE cases, by week of implementation, Tuscany, Italy, November 2018–October 2019

NDM-CRE: New Delhi metallo-beta-lactamase-producing carbapenem-resistant Enterobacterales; NW: North-West.

Health services in Tuscany, a region of 3.7 million inhabitants in the centre of Italy, are managed by the RDH in three sub-regional areas: NW with a population of 1,200,000 and 3,000 hospital beds, Central with a population of 1,500,000 and 3,000 hospital beds and South-East (SE) with a population of 800,000 and 1,600 hospital beds. Each area comprises one teaching hospital (TH), a number of district hospitals (DH) and smaller hospitals. Following the increase in NDM-CRE cases, the RDH adopted a series of measures (Figure 1) including intensified case detection activities, expanded routine screening of patients admitted to hospitals or specific hospital wards beyond usual practice (from June 2019), definition of standardised procedures for case management, data collection and infection control, and patient safety management aligned with international, national and regional guidelines [2].

**Figure 2:**
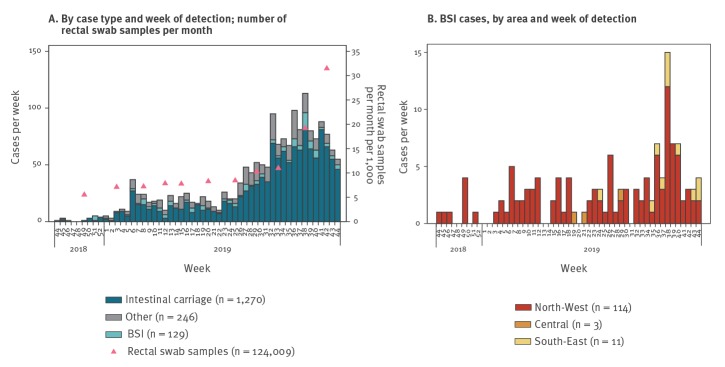
Number of tests and epidemic curve of NDM-CRE cases by week of detection, Tuscany, Italy, November 2018–October 2019 (n = 1,645)

BSI: bloodstream infection; NDM-CRE: New Delhi metallo-beta-lactamase-producing carbapenem-resistant Enterobacterales.

Before the introduction of the expanded screening programme of newly admitted patients, the region-wide average number of tests performed per month was 7,500. The number increased to 10,275 in July, 10,974 in August and 19,174 in September, reaching 31,465 in October (Figure 2A). The scale-up of molecular diagnostics use in October 2019 allowed for faster identification of NDM cases. Distribution of the screenings performed by area revealed that half of the samples (51.7%) were collected in the NW area, followed by Central (35.5%) and SE (12.8%), with the higher number in the NW area largely attributable to contact tracing activities in addition to routine screening.

Following detection of NDM-CRE carriage/infection, patients were placed under standard contact precautions. Patient and staff cohorting was implemented where necessary, depending on available infrastructure (e.g. single rooms) and number of affected patients.

## Epidemiological investigation

A case was defined as any individual referring to a healthcare facility in Tuscany as an in- or outpatient and for whom the presence of microbiologically confirmed NDM-CRE was detected in any biological sample between 1 November 2018 and 31 October 2019. Cases were classified as: intestinal carriage (isolation from surveillance rectal swabs), bloodstream infections (BSI; isolation from blood samples), colonisation or infection at other sites (isolation from the urinary or respiratory tract or other sites). Individuals with multiple isolations during the same hospitalisation episode were classified according to the most clinically relevant sample.

A dedicated standardised data collection form was developed to gather relevant information on identified cases from November 2018 onwards, including patient characteristics, hospitalisation-related data and microbiological data.

Data were collected by the laboratories and infection control teams of TH and DH and submitted weekly (cases) or monthly (screening samples) to the Regional Health Agency. Data were analysed based on time of isolation, geographical area and hospital or health facility. Cases were stratified by type, bacterial species and, for inpatients, ward or unit (intensive care units (ICU), surgical/medical wards (non-ICU), long-term care units (LTCU)). Rates were calculated as new cases occurring during the study period by total days of hospitalisation per healthcare facility, using estimates for the same period in the previous calendar year. Analyses were performed using STATA/SEv.14.2.

A total of 1,645 cases with NDM-CRE-positive microbiological samples were identified in the period from 1 November 2018 to 31 October 2019. Of these, 1,270 (77.2%) were cases of intestinal carriage, 129 (7.8%) BSI (one of them from a non-hospitalised patient), and 246 (15.0%) carriage/infection at other sites (urinary tract (180; 10.9%), other sites (66; 4.0%)) (Figure 2). Regarding sex and age distribution, 656 (41.3%) cases were male and 934 (53.7%) female (55 unknown) and the median age was 76 years (range: 0–99 years). There were differences in age distribution (Kolmogorv–Smirnov test) between male (median age: 74 years) and female cases (median age: 80 years).

Most of the cases (1,496; 90.9%) were inpatients (shown in Table), while a minority (149; 9.1%) were outpatients (e.g. community healthcare services). The majority of all cases (1,391; 84.6%) and of hospitalised cases (1,264; 84.5%) were reported in the NW area, where all TH and DH reported at least one case (data not shown), with the TH and other four DH reporting more than half of the total hospitalised cases in the area (Table). The majority of cases were hospitalised on wards other than ICU at the time of NDM-CRE detection (Table).

**Table:** NDM-CRE inpatient cases per health facility of detection, Tuscany, Italy, November 2018–October 2019 (n = 1,496)

**Area**	**Hospital type (n)**	**Confirmed NDM-CRE cases**															**Rate per 100,000 hospital days^a^** **BSI**
		**Total**	**Per case definition**						**Per ward**								
			**Intestinal carriage**		**BSI**		**Other**		**ICU**		**Other than ICU**		**LTCU**		**NA**		
			**n**	**%**	**n**	**%**	**n**	**%**	**n**	**%**	**n**	**%**	**n**	**%**	**n**	**%**	
North-West	TH (n = 1)	290	231	79.7	45	15.5	14	4.8	52	17.9	219	75.5	4	14	15	5.2	15.46
	DH (n = 4)	727	594	81.7	45	6.2	88	12.1	92	12.7	567	78.0	56	7.7	12	1.7	9.97
	Other**^b^** (n = 11)	247	178	72.1	24	9.7	45	18.2	17	6.9	108	43.7	108	43.7	14	5.7	12.84
	All facilities	1,264	1,003	79.4	114	9.0	147	11.6	161	12.7	894	70.7	168	13.3	41	3.2	11.84
Central	TH (n = 1)	26	25	96.2	1	3.8	0	0	3	11.5	23	88.5	0	0	0	0.	0.32
	DH (n = 5)	64	58	90.6	1	1.6	5	7.8	12	18.8	47	73.4	0	0	5	7.8	0.19
	Other**^b^** (n = 8)	27	25	92.6	1	3.7	1	3.7	2	7.4	19	70.4	4	14.8	2	7.4	0.64
	All facilities	117	108	92.3	3	2.6	6	5.1	17	14.5	89	76.1	4	3.4	7	6.0	0.3
South-East	TH (n = 1)	52	37	71.2	4	7.7	11	21.2	11	21.2	41	78.8	0	0	0	0.0	2.35
	DH (n = 2)	12	7	58.3	0	0	5	41.7	2	16.7	9	75.0	1	8.3	0	0.0	0
	Other**^b^** (n = 13)	51	35	68.6	7	13.7	9	17.6	4	7.8	30	58.8	17	33.3	0	0.0	3.32
	All facilities	115	79	68.7	11	9.6	25	21.7	17	14.8	80	69.6	18	15.7	0	0.0	1.9
Total (whole region)	All facilities	1,496	1,190	79.5	128	8.6	178	11.9	195	13.0	1,063	71.1	190	12.7	48	3.2	5.02

BSI: bloodstream infection; DH: district hospital; ICU: intensive care unit; LTCU: long-term care unit; NA: ward data not available; NDM-CRE: New Delhi metallo-beta-lactamase-producing carbapenem-resistant Enterobacterales; TH: teaching hospital.

**^a^** Hospitalisation days were calculated based on data for the corresponding months in the previous calendar year.

^b^ Other facilities include all hospitals not included in the former categories, e.g. primary hospitals, excluding long-term care facilities.

**Figure 3:**
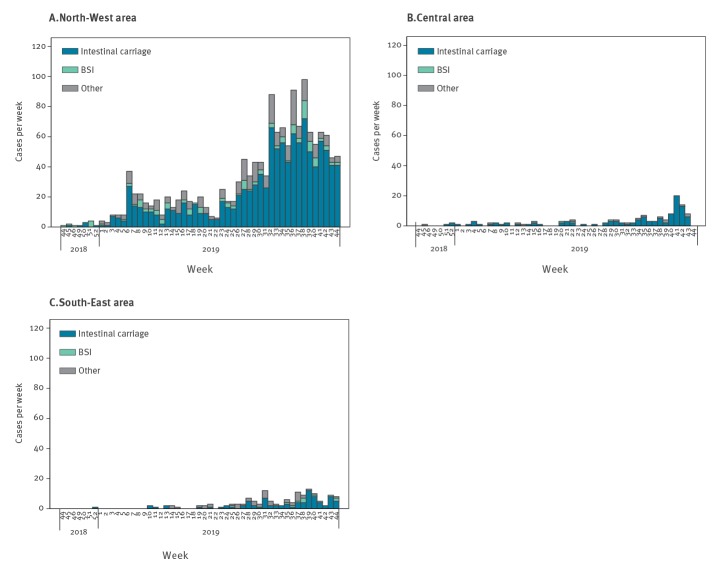
Epidemic curve, NDM-CRE cases, by week of detection and area, Tuscany, Italy, November 2018–October 2019 (n = 1,645)

According to the available data, a cluster of NDM-CRE cases occurred in November 2018 in the NW area, followed by increasing detection from mid-December 2018. The epidemic curve for the NW area shows an initial peak in mid-February 2019, followed by a further increase over the month of April 2019 (Figure 3A).

BSI: bloodstream infection.

**Figure 4:**
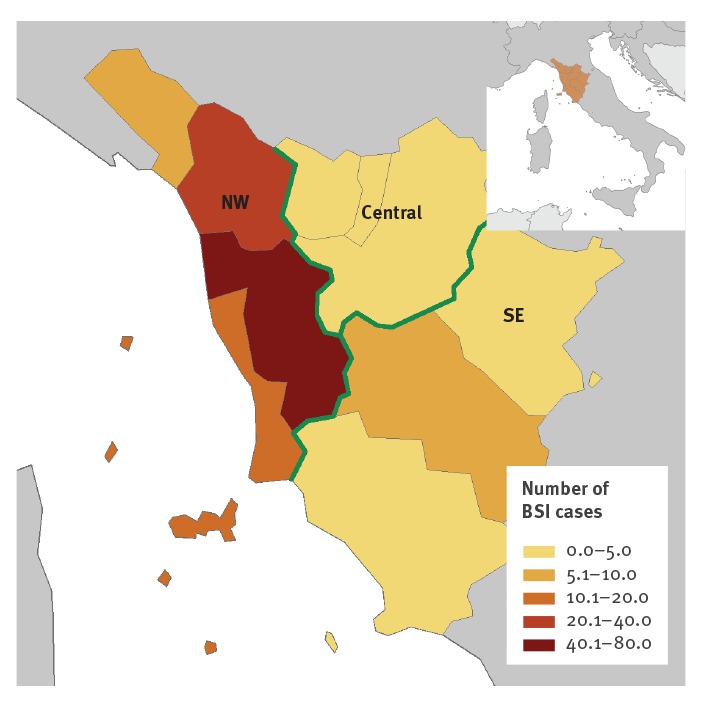
Health facilities and numbers of NDM-CRE bloodstream infections, Tuscany, Italy, November 2018–October 2019 (n = 129)

BSI: bloodstream infection; NW: North-West Tuscany; SE: South-East Tuscany.

The introduction of standardised routine screening of newly admitted patients across Tuscany in June 2019 resulted in a progressive increase in NDM-CRE detection in all TH and DH until September 2019, when a declining trend was noticed (Figures 1 and 3A). The distribution of cases over time was similar across DH in the NW area, with all facilities showing an epidemic curve with multiple peaks (data not shown). Cases in other areas of Tuscany occurred later in 2019 and were fewer (Figure 3B and C). NDM-CRE BSI cases were observed during the entire study period in the NW area, remaining sporadic elsewhere (Figures 2B and 3), as shown in the map (Figure 4). 

## Microbiological investigation

Microbiological analyses of samples were performed by the TH and DH laboratories following their standard protocols. Identification at the species level was carried out by MALDI-ToF mass spectrometry using either VitekMS (bioMérieux, Marcy-L’Etoile, France) or MALDI Biotyper (Bruker Daltonics, Bremen, Germany) or by biochemical profiling (Vitek2, bioMérieux, Marcy-L’Etoile, France). The presence and nature of carbapenemase determinants was determined by molecular testing of bacterial isolates using either PCR-based platforms (Xpert-CarbaR, Cepheid, Toulouse, France; Allplex, Seegene Inc, Seoul, Korea) or lateral flow immunochromatography systems (Resist-5 OOKNV, CorisBio, Gembloux, Belgium ). Whole genome sequencing (WGS) was performed on BSI isolates as described previously [3].

The large majority of confirmed NDM-CRE were *Klebsiella pneumoniae* (n = 1,495; 90.9%), followed by other Enterobacterales including *Escherichia coli* (n = 69; 4.2%), *Enterobacter hormaechei* (n = 2; 0.1%), *Citrobacter freundii* (n = 1; 0.1%) and unspecified NDM-CRE (n = 77; 4.7%).

Fifty-one unique NDM-positive isolates of *K. pneumoniae* from BSI were characterised by WGS. These isolates were from 12 different hospitals, mostly from the area where the outbreak had emerged. Of these isolates, 49 had been collected during the period from November 2018 to June 2019 and represented 92% of the 53 invasive isolates collected in the same period, while two had been collected earlier (July and September 2018). Most of the 51 isolates belonged to the ST147 lineage and carried the *bla*_NDM-1_ gene (n = 49; 96%), including the first two isolates collected in summer 2018. Analysis of single nucleotide polymorphisms carried out with core genomes (cgSNP) revealed that all 49 ST147 isolates were closely related to each other (cgSNP range: 1–37; mean: 16; median: 17), suggesting that clonal expansion of a single NDM-1-producing *K. pneumoniae* strain had played a major role at least in the beginning of the outbreak.

## Limitations

This study has some limitations. Heterogeneity in screening practice and coverage across the region, imperfect identification of re-admissions and sub-optimal detection of microbiologically confirmed cases before recognition of the outbreak may have affected the accuracy of the present assessment. Furthermore, the reliance on paper-based data collection systems in some facilities and lengthy data collation and validation processes hampered our capacity to extend the present study period beyond week 44 of 2019. While the molecular characterisation data presented in this study are representative of the early phase of the outbreak, further analyses are needed to characterise invasive isolates collected in the second half of 2019.

## Public health implications and conclusions

NDM-CRE are able to hydrolyse almost all beta-lactams and are not inhibited by currently available beta-lactamase inhibitors, including the new ones avibactam and vaborbactam [4]. NDM-CRE were first described in 2008 in Sweden and subsequently reported across Europe [1,5], resulting in growing concern for these pathogens to become endemic in the region [6,7]. In Italy, a country where CRE are reported at endemic level [8], NDM-CRE were first detected in 2009 [9,10] and sporadic, often travel-related, cases have been recorded since [5]. This was also the case in Tuscany until November 2018, when a noticeable increase of NDM-CRE isolates was registered [1].

This is a report of a large and persistent outbreak in an Italian region, probably caused by a single-clone NDM-CRE, highlighting the risk of rapid emergence and disseminations of uncommon variants of CPE within healthcare facility networks, as recently reported in other European countries [6,7]. Tuscany has a comprehensive and effective microbiological surveillance system, yet the increase in NDM-CRE cases was detected with some delay. This was probably due to the following circumstances: (i) Italy is a setting of high endemicity for CRE and occurrence of CRE isolates was not unexpected, (ii) the routine surveillance system is based on phenotypic resistance profiles to various indicator antibiotics, not including those suggestive of NDM-CRE emergence (e.g. ceftazidime-avibactam), (iii) CRE resistance mechanisms were not routinely searched and (iv) data integration and analysis in the laboratory information systems are not automatised.

Delayed outbreak identification compromised the implementation of a rapidly effective response to contain NDM-CRE spread across healthcare facilities in the NW area. However, the set-up of a regional RTF and the coordinated roll-out of a comprehensive bundle of interventions was successful in preventing the spread within the Central and SE areas. During the outbreak, infection control protocols were streamlined, combining the contact precautions protocols that were already present with organisational improvements such as cohorting of staff and patients. Most of the efforts were directed towards early case finding and expanding screening protocols in all wards and health facilities. Before the outbreak, routine screening with rectal swabs was performed heterogeneously across Tuscany and largely targeted towards patients admitted to ICU or towards immunocompromised patients, e.g. patients admitted to a haematology ward.

The three sub-regional areas have a nearly closed patient referral system, with patients circulating between the TH and DH/other smaller hospitals located within each area. This referral pathway may have enhanced circulation of NDM-CRE through a combination of different mechanisms. While intra-hospital transmission has probably been substantial, inter-hospital and community-to-hospital circulation through patients navigating the health system with multiple admissions across different health facilities are probable reasons for the geographical spread [11,12], at least within the NW area [13]. However, the same self-sufficiency of the Health Services within each area is likely to have contributed to confining the circulation of cases within their borders.

## Conclusion

The emergence of NDM-CRE strains in Tuscany, where CRE *K. pneumoniae* circulation within healthcare facilities is already sustained [14], is of great concern as therapeutic options remain very limited. Despite the observed decreasing trend in the number of new cases detected since October 2019, continued monitoring of NDM-CRE transmission is required to assess the risk of further spread within and beyond Tuscany region.
